# A de novo* PAK1* likely pathogenic variant and a de novo terminal 1q microdeletion in a Chinese girl with global developmental delay, severe intellectual disability, and seizures

**DOI:** 10.1186/s12920-023-01433-x

**Published:** 2023-01-09

**Authors:** Jianlong Zhuang, Meihua Xie, Jianfeng Yao, Wanyu Fu, Shuhong Zeng, Yuying Jiang, Yuanbai Wang, Yingjun Xie, Gaoxiong Wang, Chunnuan Chen

**Affiliations:** 1 Prenatal Diagnosis Center, Quanzhou Women’s and Children’s Hospital, Quanzhou, 362000 People’s Republic of China; 2 Prenatal Diagnosis Center, Yueyang Central Hospital, Yueyang, 414000 People’s Republic of China; 3 Department of Women Healthcare, Quanzhou Women’s and Children’s Hospital, Quanzhou, 362000 People’s Republic of China; 4 Department of Obstetrics and Gynecology, Guangdong Provincial Key Laboratory of Major Obstetric Diseases, Key Laboratory of Reproduction and Genetics of Guangdong Higher Education Institutes, Third Affiliated Hospital of Guangzhou Medical University, Guanghzou, 510150 People’s Republic of China; 5 Key Laboratory of Reproduction and Genetics of Guangdong Higher Education Institutes, Third Affiliated Hospital of Guangzhou Medical University, Guangzhou, 510150 People’s Republic of China; 6 Quanzhou Women’s and Children’s Hospital, Quanzhou, 362000 People’s Republic of China; 7 Department of Neurology, The Second Affiliated Hospital of Fujian Medical University, Quanzhou, 362000 Fujian Province People’s Republic of China

**Keywords:** 1q44 microdeletion, Chromosomal microarray analysis, Whole exome sequencing, Developmental delay, Seizures, Intellectual disability

## Abstract

**Background:**

Pathogenic *PAK1* variants were described to be causative of neurodevelopmental disorder with macrocephaly, seizures, and speech delay. Herein, we present a de novo* PAK1* variant combine with a de novo terminal 1q microdeletion in a Chinese pediatric patient, aiming to provide more insights into the underlying genotype–phenotype relationship.

**Methods:**

Enrolled in this study was a 6-year-old girl with clinical features of global developmental delay, severe intellectual disability, speech delay, and seizures from Quanzhou region of China. Karyotype and chromosomal microarray analysis (CMA) were performed to detect chromosome abnormalities in this family. Whole exome sequencing (WES) was performed to investigate additional genetic variants in this family.

**Results:**

No chromosomal abnormalities were elicited from the entire family by karyotype analysis. Further familial CMA results revealed that the patient had a de novo 2.7-Mb microdeletion (arr[GRCh37] 1q44(246,454,321_249,224,684) × 1]) in 1q44 region, which contains 14 OMIM genes, but did not overlap the reported smallest region of overlap (SRO) responsible for the clinical features in 1q43q44 deletion syndrome. In addition, WES result demonstrated a de novo NM_002576: c.251C > G (p.T84R) variant in *PAK1* gene in the patient, which was interpreted as a likely pathogenic variant.

**Conclusion:**

In this study, we identify a novel *PAK1* variant associated with a terminal 1q microdeletion in a patient with neurodevelopmental disorder. In addition, we believe that the main clinical features may ascribe to the pathogenic variant in *PAK1* gene in the patient.

## Introduction

The p21-activated kinases (PAKs) are a family of serine/threonine kinases consist of six members (PAK1-6), which are active upon Rho GTPases that regulate several signal pathways including Ras/Raf/MEK/ERK and Wnt/β-catenin, and other underlying pathways [1, 2]. The PAKs family can be divided into group I (PAK1, PAK2 and PAK3) and group II (PAK4, PAK5 and PAK6) based on domain architecture and regulation [3]. PAKs have been implicated in several human disorders, variants in *PAK3* gene have been described in males with X-linked recessive developmental delay (OMIM: 300558) [4]. The *PAK1* (OMIM: 602590) gene located in 11q13.5q14.1 region is highly expressed during embryogenesis and in adult tissues including the brain, muscle, and spleen [5]. In the recent reports, de novo* PAK1* variants were described to be causative of neurodevelopmental disorder with macrocephaly, seizures, and speech delay [6].

Chromosome terminal 1q microdeletion is a less common chromosome syndrome with only sporadic cases available in the literature, which were commonly identified as interstitial and terminal deletions. Patients with chromosome terminal 1q microdeletion syndrome commonly exhibit developmental delay, intellectual disability, microcephaly, craniofacial anomalies, seizures, and abnormality of the corpus callosum [7–9]. Interestingly, previous studies indicated three distinct smallest regions of overlap (SRO), which demonstrated different sizes in the 1q43q44 microdeletion region. The first region is a ~ 75 kb fragment in size including the *ZNF238* gene and responsible for corpus callosum abnormalities; the second region contains the *AKT3* gene that responsible for microcephaly; the last region is a ~ 100 kb fragment that overlaps *HNRNPU*, *FAM36A* and *NCRNA00201* genes and is proposed to be the candidate region for seizures [10–12].

In the study, we present a new de novo terminal 1q microdeletion in a Chinese pediatric patient who manifested developmental delay, speech delay, severe intellectual disability, and seizures, without covering the three distinct SRO in 1q43q44 microdeletion region. In addition, an additional de novo variant in *PAK1* gene was identified in the patient using whole exome sequencing.

## Material and methods

### Subjects

Enrolled in this study was a family from Quanzhou region Fujian province of China. This family denied consanguineous marriage and any familial inherited diseases. Karyotype, chromosomal microarray analysis and whole exome sequencing were carried out for chromosomal abnormalities and genetic variants detection in the family after signed the written inform. Ethics Committee approval was obtained from the Institutional Ethics Committee of Quanzhou Women’s and Children’s Hospital to the commencement of the study (2020No.31).

### Karyotype analysis

Approximately 2–3 ml peripheral blood were collected from the patient and the parents for karyotype analysis. The peripheral blood lymphocytes were harvested using a SinochromeChromprepII automatic chromosome harvesting system according to the standard protocol (Shanghai Lechen Biotechnology Co., Ltd.), which has been described previously in our study [13]. After staining with Giemsa stain, twenty karyotypes were counted and analyzed five karyotypes.

### Genomics DNA extraction

About 3–5 ml peripheral bloods were collected from the patient and the parents for chromosomal microarray analysis, Sanger sequencing and whole exome sequencing. Genomics DNA were extracted from enrolled members’ peripheral blood using QIAamp DNA Blood Kit (QIAGEN, Germany) according to the manufacturer’s protocol (www.qiagen.com).

### Chromosomal microarray analysis

Chromosomal microarray analysis was performed using single-nucleotide polymorphism based Affymetrix Cytoscan 750 K chip (Life Technologies, American) according to the protocol described previously [14]. Copy number variants (CNVs) were further assessed according to Database of Genomic Variants (DGV), Online Mendelian Inheritance in Man (OMIM), DECIPHER and PubMed databases, as well as other databases and our local database. The CNVs pathogenicity interpretation was conducted according to a joint consensus of the American College of Medical Genetics (ACMG) and the Clinical Genome Resource (ClinGen) standards and guidelines [15].

### Whole exome sequencing and data analysis

The genomics DNA in the enrolled family were further subjected to WES analysis. DNA quantification was carried out using the Qubit dsDNA HS Assay (Invitrogen, Carlsbad, CA, USA). Approximate mean fragment length of 150–200 bp were sheared using the Covaris LE220 (Covaris, Woburn, MA, USA). Then, the sheared DNA were used for library preparation of targeted regions by SureSelect whole-exome capture kit (Agilent). The Illumina DNA Standards and Primer Premix Kit (Kapa Biosystems, Boston, MA, USA) was used for the sequencing libraries quantification. Subsequently, the Illumina HiSeq 2500 platform (Illumina, San Diego, CA, USA) was used for sequencing.

The specific process of data analysis was conducted according to the description of our previous study [16]. Data analysis was processed included variant calling, annotation and variant screening. The dbSNP, 1000 Genomes Project, Exome Aggregation Consortium and Exome Variant Server databases were used for searching the minor allele frequencies (MAF < 0.1%) of all known variants. The OMIM, ClinVar, Human Gene Mutation Database and SwissVar databases were used to determine the harmfulness and pathogenicity of the detected variants. Variants were classified as pathogenic, likely pathogenic, variants of unknown significance (VOUS), likely benign and benign, according to the ACMG guidelines [17]. Sanger sequencing was further performed for verification of the variants detected by WES.

## Results

### Subject information

Recruited in this case report was a 6-year-old girl, who was the first child of the family. Both of her parents were 30-year-old, who denied consanguineous marriage and any history of familial genetic diseases. She was born naturally at the gestational age of 39^+4^ weeks, with 3.0 kg (+ 0.2 SD) in birth weight and a 50 cm (− 0.6 SD) in height. No threatened abortion or prenatal ultrasound anomalies were observed during the pregnancy. However, an obvious developmental milestone delay was observed, she was unable to sit independently at 18 months, could crawl at 26 months, and walk independently at over 4 years of age. A subsequent children psychological test elicited a low intelligence quotient (scores: 19), based on which a diagnosis of severe intellectual disability was made.

At the age of 3^+^ years, seizures occurred, accompanied with loss of consciousness, clenched fists, twitching limbs, without foaming at the mouth and fever. Seizures could relieved spontaneously in about one minute, with a daily frequency of 4–5 episodes. The symptom could be controlled by oral administration of antiepileptic drugs. Electroencephalography (EEG) showed abnormal brain waves, including synchronous paroxysmal slow wave rhythm with high amplitude, asymmetric amplitude on both sides, and overlapping sharp slow waves during the period, which was obvious in both frontotemporal areas. No abnormalities were observed in brain MRI detection. She is now 6 years and 10 months old with 115 cm (− 1.4 SD) in height and 23 kg (+ 0.2 SD) in weight. But she is unable to speak and defecate by herself, and she could only understand simple instructions. In addition, physical examination showed that she had normal consciousness, and limb muscle strength was defined as class V. She had a normal head circumference, with no significant deformities in the hands and feet, but mild facial abnormalities were observable including ocular hypertelorism, flat nasal bridge, irregular teeth, and hydrostomia. Later, the couple gave birth to a girl and a boy in 2018 and 2021, respectively, both showing normal clinical features and developmental milestones.

### Karyotype and chromosomal microarray analysis results

**Fig. 1:**
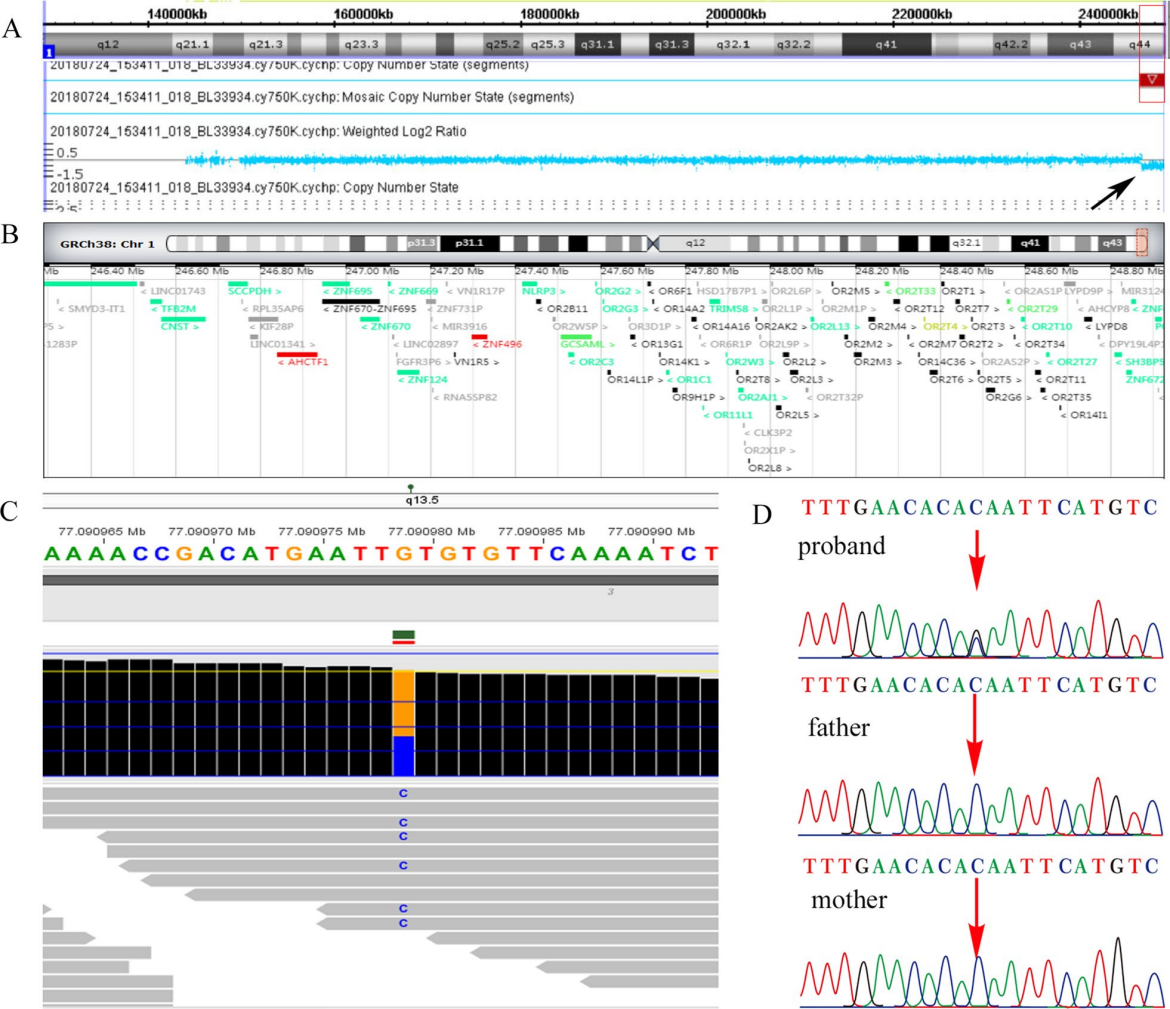
The result of chromosomal microarray analysis and whole exome sequencing in the patient. **A** A 2.7-Mb deletion in 1q44 region as detected by chromosomal microarray analysis. As shown in **B**, the terminal deletion of 1q44 contained 14 OMIM genes, including *SMYD3, TFB2M, CNST, AHCTF1, ZNF695, ZNF124, ZNF496, NLRP3, OR13G1, OR2W3, OR2M7, OR14I1, LYPD8,* and *ZNF692*. **C** A novel c.251C > G (p.T84R) variant in *PAK1* gene was identified in the patient by WES technology. **D** Sanger sequencing results confirmed the c.251C > G variant in the patient, and no relevant variant was observed in her parents

No chromosomal abnormality was detected in the entire family by karyotype analysis. The subsequent CMA result demonstrated that the patient had a 2.7-Mb deletion (arr[GRCh37]1q44(246454321–249224684) × 1) in 1q44 region, containing 14 OMIM genes including *SMYD3, TFB2M, CNST, AHCTF1, ZNF695, ZNF124, ZNF496, NLRP3, OR13G1, OR2W3, OR2M7, OR14I1, LYPD8,* and *ZNF692* (Fig. 1). No CNVs were detected in the parents and the other two siblings, suggesting that the 1q44 deletion in the patient was a de novo variant and was interpreted as variant of uncertain significance according to the ACMG guidelines. In addition, partial cases of 1q44 microdeletion reported in the literature were reviewed and listed in Table 1.

**Table 1:** Clinical findings and the encompassed genes in patients with terminal 1q44 microdeletion

	Thierry et al. [12]	Cho et al. [18]	Westphal et al. [19]	Tung et al. [20]	Caliebe et al. [21]	Raun et al. [22]	Selmer et al. [23]	Gupta et al. [24]	Perlman et al. [25]	Our study
Deletion size	0.63–2.56 Mb	NM	1.2 Mb	28.7 Kb	1.1–9.43 Mb	4.1 Mb	163 Kb	1.8 Mb	1.47 Mb	2.7 Mb
Inheritance	de novo (10/11)	de novo	de novo	de novo	de novo (3/4)	de novo	de novo	NM	de novo	de novo
Sex/Age(years)	0.5–3	F/2	F/1.5	F/5	1.25–11	M/8	M/10	F/3	F/2	F/6
Global developmental delay	11/11	+	+	+	4/4	+	+	+	+	+
Intellectual disability	11/11	+	NM	+	4/4	+	+	NM	NM	+
Seizures	11/11	−	+	+	4/4	+	+	+	+	+
Speech delay	7/11	NM	NM	+	4/4	+	+	+	+	+
Microcephaly	2/11	+	+	−	3/4	+	−	−	−	−
Abnormal corpus callosum	1/11	+	+	+	4/4	+	+	+	+	−
Short stature	5/11	NM	NM	+	2/4	NM	+	+	+	−
Facial abnormality	11/11	+	+	+	4/4	+	+	+	+	+
Other	Autistic features, obesity, small hands and feet, etc	Supernumerary nipple and auricular pit, polydactyly in foot	Postaxial hexadactyly	Right smallest toe was duplicated	Hypotonia	Autonomic abnormalities	Autism, hypotonia and a bifid uvula	Preaxial polydactyly, hemiparesis of right side	Small hands and feet	−
Gene content	Smallest region of overlap HNRNPU, FAM36A and NCRNA00201	NM	ZNF238, ADSS, DESI2, COX20, HNRNPU, and KIF26B	COX20 and HNRNPU	Smallest region of overlap FAM36A, HNRPU, the EFCAB2 genes and part of the KIF26B	Covering HNRNPU, SMYD3, NLRP3, and KIF26B, etc	FAM36A and HNRNPU	C1orf101, PPPDE1, FAM36A, NCRNA00201, HNRNPU, EFCAB2, KIF26B, and SMYD3	Covering ZNF238, etc	SMYD3, TFB2M, CNST, AHCTF1, ZNF695, ZNF124, ZNF496, NLRP3, OR13G1, OR2W3, OR2M7, OR14I1, LYPD8 and ZNF692

F: female; M: male; −: absent; + : present; NM: not mentioned

### Whole exome sequencing results

WES technology was further employed to investigate additional variants in the patient using peripheral blood. A novel NM_002576: c.251C > G (p.T84R) variant in *PAK1* gene in the patient and verified by Sanger sequencing (Fig. 1). Parental sanger sequencing verification indicated that the novel variant observed in the patient was de novo (Fig. 1) (PM6). No frequency was observed in the databases of gnomAD, 1000 genomes, dbSNP, PubMed, HGMD and ClinVar (PM2_Supporting). According to the online computer-aided analysis predictions (http://159.226.67.237/sun/varcards/welcome/index) the c.251C > G variant was predict to affect protein structure/function (Damaging scroe: 0.83) (PP3). In addition, the c.251C > G variant in *PAK1* gene located in the p21-Rho-binding domain, which binding Cdc42p- and/or Rho-like small GTPases according to the UCSC database (PM1). Furthermore, the patient’s clinical presentation is consistent with this gene evaluated by clinical experts (PP4). Finally, the variant was interpreted as likely pathogenic variant according to the ACMG guidelines (PM1, PM6, PM2_Supporting, PP3, PP4).

## Discussion

In the clinical practice, CMA technology manifests a great advantage in copy number variants (CNVs) detection, as well as uniparental diploid and triploid, and it has been recommended as a first-line detection tool in etiological diagnosis of patients with multiple congenital anomalies [26, 27]. In addition, WES technology has been recommended as a fundamental tool to investigate additional sequence variants in patient with normal CMA result or unexplained CNVs [28, 29]. In the present study, we present a Chinese pediatric patient with global developmental delay, speech delay, severe intellectual disability, and seizures and had a de novo* PAK1* gene variant associated with a de novo terminal 1q44 microdeletion, without covering the reported three distinct SRO in 1q43q44 microdeletion region.

Causative variants in *PAK1* gene were related to intellectual developmental disorder with macrocephaly, seizures, and speech delay (IDDMSSD; 618,158). To date, extremely rare reports of *PAK1* variants that result in IDDMSSD are available in the literature. Additionally, most of the patients had de novo* PAK1* variants [6]. The PAK1 are activated upon binding the GTP-bound forms of the Rho GTPases *CDC42* (OMIM: 116,952) and *RAC1* (OMIM: 602,048). In addition, pathogenic variants in *RAC1* and *CDC42* genes are associated with developmental disorders [30]. A previous study reported two unrelated subjects who had de novo c.392A > G (p.Tyr131Cys) and c.1286A > G (p.Tyr429Cys) variants in *PAK1* gene exhibited developmental delay, macrocephaly, seizures, and ataxic gait [6]. Both patients’ fibroblasts showed increased phosphorylation of downstream PAK1 targets and a trend of increased PAK1 kinase activity, which indicating a gain of function effect of the variants. In addition, gain of function mechanism of *PAK1* variant was also supported that knockout of either *PAK1* or *PAK3* in mice results in no obvious abnormalities [31]. In addition, a previous study [32] present a patient with neurodevelopmental disorder, seizures, and macrocephaly caused by a de novo p.Ser110Thr missense variant in *PAK1* gene, indicating the important role of *PAK1* in controlling postnatal brain development and volume. However, a previous study conducted by Horn et al. [30] presented four patients who harbored *PAK1* gene variants with intellectual disability, macrocephaly and seizures, with one of them did not manifests macrocephaly.

In our case report, a de novo c.251C > G (p.T84R) variant in *PAK1* gene was also identified using WES technology who had similar features including global developmental delay, severe intellectual disability, speech delay, and seizures, without macrocephaly. Thus, the clinical feature of macrocephaly may manifest incomplete penetrance in patients with *PAK1* variants. As for the importance of *PAK1* in neuronal growth and structure, as well as the previous reported cases, we believe that the de novo* PAK1* gene variant in the patient may responsible for the major clinical features of neurodevelopmental disorder. However, we can not rule out the pathogenic of 1q44 microdeletion that contributing to the phenotypes such as developmental delay and intellectual disability.

Although the terminal 1q microdeletion in our study did not covering the reported three distinct SRO in 1q43q44 microdeletion region, several OMIM genes including *SMYD3* were contained. As demonstrated in the DECIPHER database, two cases with 1q44 microdeletion (DECIPHER ID: 338648 and 426113) only containing *SMYD3* gene exhibited global developmental delay, intellectual disability, seizures, and stereotypy. SMYD3 is a histone methyltransferase, playing a role in transcriptional regulation as a RNA polymerase complex, and also an important role in carcinogenesis and metastasis [33]. A previous study conducted by Wang et al. [34] indicated that the deletion of *SMYD3* was responsible for the intellectual disability phenotype in their cases. Thus, the *SMYD3* deletion may also responsible for partial clinical features in this study.

In conclusion, our study presented a patient with terminal 1q microdeletion with global developmental delay, severe intellectual disability, speech delay, and seizures, without covering the reported three distinct SRO in 1q43q44 microdeletion region. Interestingly, an additional c.251C > G (p.T84R) variant in *PAK1* gene was identified, which may be the main reason for the patient’s clinical phenotypes. Moreover, our study also strengthened the application value of CMA and WES in the etiologic diagnosis of in patients with unexplained congenital abnormality.

## Data Availability

The datasets used and analyzed in the current study were obtained from the corresponding author on reasonable request.
